# Biogenic copper nanoparticles from *Avicennia marina* leaves: Impact on seed germination, detoxification enzymes, chlorophyll content and uptake by wheat seedlings

**DOI:** 10.1371/journal.pone.0249764

**Published:** 2021-04-15

**Authors:** Hanaa L. Essa, Mohamed S. Abdelfattah, Alaa S. Marzouk, Zeinab Shedeed, Hania A. Guirguis, Mayyada M. H. El-Sayed

**Affiliations:** 1 Chemistry Department, American University in Cairo, New Cairo, Egypt; 2 Pesticides Phytotoxicity Department, CAPL, Agriculture Research Centre, Dokki, Giza, Egypt; 3 Natural Products Research Unit (NPRU), Chemistry Department, Faculty of Science, Helwan University, Ain Helwan, Cairo, Egypt; 4 Botany and Microbiology Department, Faculty of Science, Helwan University, Ain Helwan, Cairo, Egypt; VIT University, INDIA

## Abstract

Biogenic copper nanoparticles (Cu NPs) were synthesized using the aqueous crude extract of mangrove leaves, *Avicennia marina* (CE). GC-MS metabolite profiling of CE showed that their carbohydrates are mainly composed of D-mannose (29.21%), D-fructose, (18.51%), L-sorbose (12.91%), D-galactose (5.47%) and D-Talose (5.21%). Ultra-fine nanoparticles of 11.60 ±4.65 nm comprising Cu_2_O and Cu(OH)_2_ species were obtained with a carbohydrate and phenolic content of 35.6±3.2% and 3.13±0.05 mgGA/g, respectively. The impact of the biogenic Cu NPs on wheat seedling growth was dose-dependent. Upon treatment with 0.06 mg/mL of Cu NPs, the growth was promoted by 172.78 ± 23.11 and 215.94 ± 37.76% for wheat root and shoot, respectively. However, the lowest relative growth % of 81.94 ± 11.70 and 72.46 ± 18.78% were recorded for wheat root and shoot, respectively when applying 0.43 mg/mL of Cu NPs. At this concentration, peroxidase activity (POX) of the germinated wheat seeds also decreased, while ascorbic acid oxidase (AAO) and polyphenol oxidase (PPO) activities increased. Higher uptake of copper was observed in the root relative to the shoot implying the accumulation of the nanoparticles in the former. The uptake was also higher than that of the commercial Cu NPs, which showed an insignificant effect on the seedling growth. By treating the wheat leaves in foliar application with 0.06 mg/mL of Cu NPs, their contents of Chlorophyll a, Chlorophyll b, and total chlorophyll were enhanced after 21 days of application. Meanwhile, the high concentration (0.43 mg/mL) of Cu NPs was the most effective in reducing the leaf content of chlorophyll (a, b, and total) after the same time of application. The findings of this study manifest the potential of utilizing controlled doses of the prepared biogenic Cu NPs for inhibition or stimulation of seedling growth.

## 1. Introduction

Marine plants have been recently the subject of a myriad of studies due to their diverse biological activities. One plant of interest is the mangrove, which grows in small trees along tropical and sub-tropical coasts. Asia has the largest amount (42%) of the world’s mangroves, followed by Africa (21%), Northern, Central America and the Caribbean (15%), Oceania (12%), and South America (11%) [1]. Two of the world’s mangrove species exist in Egypt, and are commonly known as gray and red mangroves or scientifically as *Avicennia marina* and *Rhizophora mucronata*, respectively [2].

The former species shortly referred to as *A*. *marina*, is more abundant in Egypt as it thrives along the Red Sea Coast lining from Ras Mohamed to Mersa-Halaib [3]. Nowadays, several biological activities have been reported for mangroves such as antimicrobial [4], antioxidant [5], antidiabetic [6], antibacterial [7] and anticancer activities [8], and these were owed to its composition, which constitutes phytochemicals as alkaloids, phenolic compounds, steroids, terpenoids, glucosides and flavonoids together with polysaccharides [9–11]. In addition to their bioactivities, the mangrove extracts can be utilized as reducing agents for the synthesis of metal/metal oxide nanoparticles (NPs) that are bioactive, probably due to their unique physicochemical properties that are related to their high surface area, high reactivity, tunable pore size, and particle morphology. Along with their role in bioreduction, the extracts form a stabilizing layer around the NPs, preventing them from agglomeration and contributing to their bioactivity through its active functional groups.

Biosynthesized NPs have been used in agriculture as fertilizers, insecticides, herbicides and fungicides [12–14]. To evaluate the bioactivity of NPs for these applications, a seed germination phytotoxicity test is usually conducted. Germination generally refers to seeds’ emergence and seedling growth of seedling shoots and root length [15].

One of the biosynthesized NPs that have not been extensively studied for their impact on seed germination is Cu NPs, which is the focus of our work. In an earlier study, the germination of lettuce (*L*. *sativa*) seeds in aqueous medium containing Cu NPs showed that CuO NPs were slightly more toxic than their Cu_2_O counterparts and this consequently led to reduction of seed germination and root elongation [16]. Another study showed that *Cucumis sativus* Cu NPs accumulated in the roots of the cucumber plant and consequently reduced root length and root biomass [17]. The reported toxicity of Cu NPs could stem from their chemical composition that might lead to release of toxins and/or the stress stimulation they might cause due to their size, shape or surface [12].

**Fig 1:**
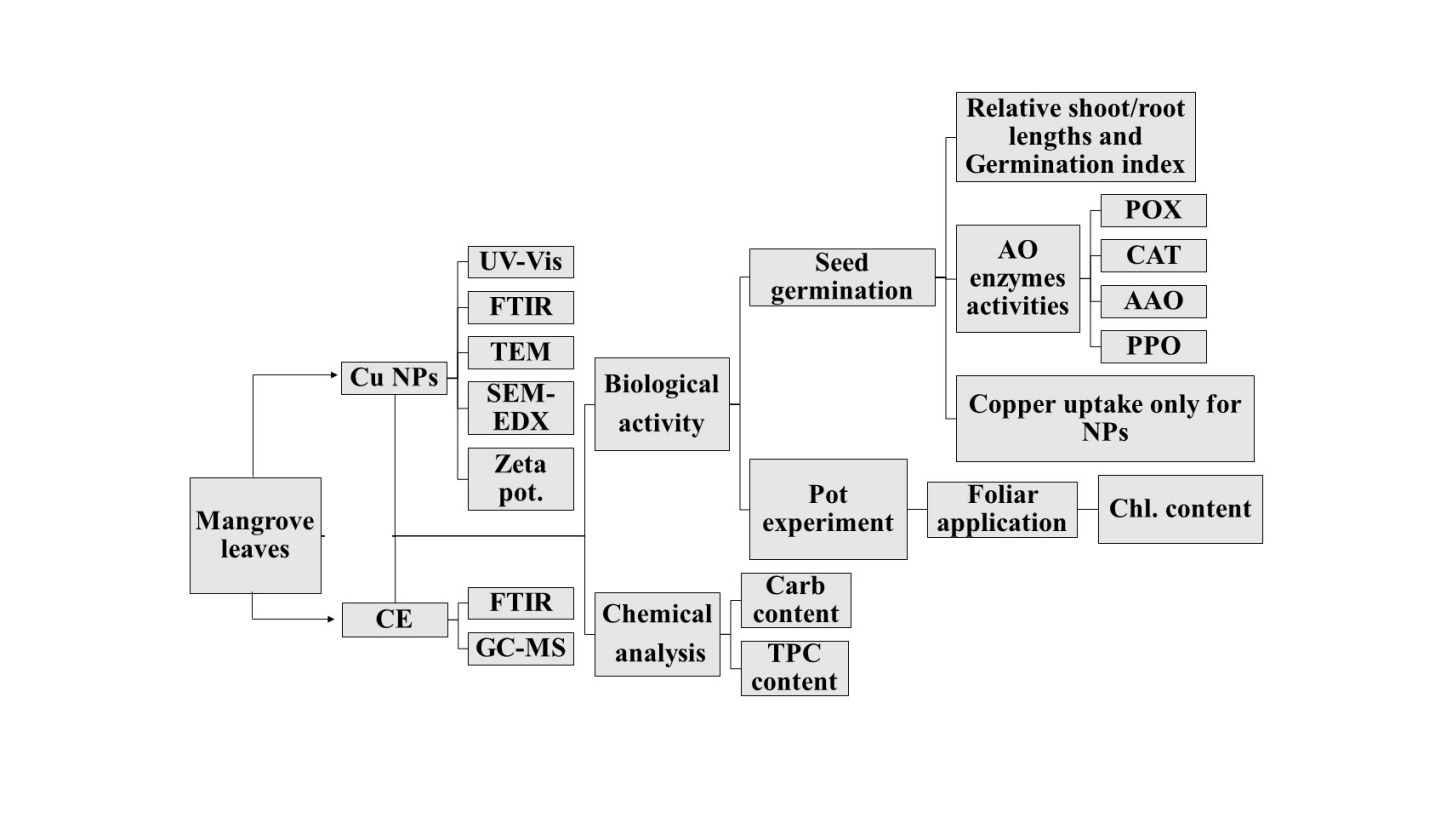
Schematic outline of the work conducted in this study. FTIR: Fourier Transform Infrared Spectroscopy, GC-MS: Gas Chromatography-Mass Spectrometry, XRD: X-ray Diffraction, EDX: Energy-dispersive X-ray spectroscopy, TEM: Transmission Electron Microscopy, SEM: Scanning Electron Microscopy, UV-Vis: Ultraviolet-Visible spectrophotometry, Carb: Carbohydrates, TPC: Total Phenolic Content.

This work aims to utilize a new green method for synthesizing biogenic Cu NPs using the crude extract (CE) of *A*. *marina* as a reducing agent. The proposed synthesis method readily forms NPs that are coated by a capping layer of CE with active functional groups which can potentially affect plant growth. To determine the conditions necessary for the biogenic Cu NPs to function as either growth inhibitors or stimulants, their effect on the germination of wheat seeds, grown in petri dishes, was investigated under different NPs concentrations and was compared with that of commercial Cu NPs as well as that of CE. The germinated wheat seedlings were tested for their copper uptake, while their detoxification enzymes were studied to examine the phytotoxicity effect of the biogenic NPs and the CE on them. In addition to the seed germination studies, pot experiments were conducted where the wheat seeds were planted for 14 days after which a foliar spray of CE or CuNPs (commercial and biogenic) was applied. Fig 1 depicts the outline of this work.

## 2. Materials and methods

### 2.1. Chemicals

For chemical analyses, phenol (BDH, England), sulfuric acid of concentration 98% (Penta, Prague, Czech Republic) and Folin & Ciocalteu’s phenol reagent (Loba Chemie, Colaba, Mumbai, India) were used. For nanoparticle synthesis, copper sulfate pentahydrate (CuSO₄.5H₂O) was purchased from Anachemia Ltd. (Montreal, Canada) and commercial CuO nanoparticles (size < 50±7 nm as detected by TEM) were purchased from NanoTech (Cairo, Egypt). For pot experiment, the fertilizer NPK (20/20/20) was purchased from Misr El-Dawliya for Agricultural & Industrial Development Co. (Kwesna, Egypt) and dimethyl sulfoxide (DMSO) was purchased from Daejung Chemical & Metal Co., Ltd (Korea).

### 2.2. Sample collection

Fresh mangrove leaves (*Avicennia marina* (Forssk.) Vierh), were collected from Ras Mohammed national park at southern Sinai coast (Gulf of Aqaba) during Winter 2017. Voucher specimens of the plant were identified by Dr. Mohamed Massed Hejazi in the Botany Herbarium lab of Marine Science Department, Suez Canal University, Ismailia, Egypt. Leaves were transferred into moist plastic bags, air-dried and ground in an electric mortar, then powdered samples were stored in the refrigerator at -20 ^0^C for future use.

### 2.3. Aqueous crude extraction

Five grams of ground *A*. *marina* leaves were suspended in 100 mL of distilled water. The suspension was heated for 20 min at 70°C and subsequently filtered through a cheesecloth. The filtrate was stored in the refrigerator for future use. Extraction was performed in triplicates and the percent yield was 35.2% ± 1.5.

### 2.4. Gas Chromatography-Mass Spectrometry (GC-MS) analysis of the silylated primary metabolites in the crude extract

Primary metabolites analysis was carried out as follows. Briefly, 20 mg of extract were dissolved with 5 mL of 100% methanol under sonication for 5 min with frequent shaking, followed by centrifugation at 12,000 x g for 10 min. A volume of 100 μL of the methanolic extract was aliquoted in screw-cap vials and left to evaporate under a nitrogen gas stream until complete dryness. For derivatization, 150 μL of N-methyl-N-(trimethylsilyl)-trifluoroacetamide (MSTFA) that was previously diluted to 1/1% with anhydrous pyridine was added to the dried methanol extract and incubated at 60°C for 45 min prior to analysis using GC-MS. Separation of silylated derivatives was accomplished on a low-polarity fused silica column, Rtx-5MS (30 m length, 0.25 mm inner diameter, and 0.25 m film) [18]. Identification of silylated components was performed by comparing their retention indices (RI) relative to n-alkanes (C6- C20), and their mass spectral matching to that of the National Institute of Standards and Technology (NIST) in Maryland, USA; as well as by using WILEY library database and standards when available. The separation of the compounds onto the column was based on differences in their volatility and polarity. Peaks were first deconvoluted using AMDIS software (2.70, NIST, Gaithersburg, MD, USA, 2011) prior to mass spectral matching.

### 2.5. Synthesis of the biogenic copper nanoparticles

In a 250 mL Erlenmeyer flask, 10 mL of *A*. *marina* leaf extract was added to a 100-mL solution of 4 mM of copper sulfate pentahydrate (CuSO_4_.5H_2_O) and the mixture was kept stirring for 3 h at 70°C until its color changed from blue to green [19, 20]. The formation of the biogenic Cu NPs was confirmed and its absorbance was monitored along with that of the commercial CuO nanoparticles using a UV-Vis spectrophotometer (Varian, Cary 500 Scan, Palo Alto, California, USA). To obtain pure nanoparticles; the nano-solution was centrifuged (Heraeus-Christ, GMBH336 Osteode Ma Harz No.39189, Hanau, Germany) for 10 min at -10°C / 5500–6000 rpm. The precipitate was discarded and the supernatant was dialyzed to get rid of the unreacted copper sulfate and obtain the purified nano-suspension.

### 2.6. Characterization of the biogenic copper nanoparticles

#### 2.6.1. Fourier Transform Infrared (FTIR) spectroscopic analysis

CE and Cu NPs (biogenic and commercial) were mixed with potassium bromide to form 1-mm pellets. Analysis was carried out using a Nicolet 380 Thermogravimetric Analysis/Fourier Transform Infrared (TGA/FTIR) spectrometer range of 500 to 4000 cm^-1^ wavenumbers.

#### 2.6.2. Transmission and Scanning Electron Microscopy with elemental analysis

The size and surface structure of the biogenic nanoparticles were examined using Transmission Electron Microscopy, TEM (Type JEOL-JEM-2100, Software: Gatan Digital Micrograph, Akishima, Tokyo, Japan). Specimens of a few mgs of powdered samples were suspended in distilled water and were well dispersed in an ultrasonic bath for 15 min. Few drops of the suspension were poured onto previously prepared grids covered by a thin film of evaporated carbon, and then the prepared grids were examined under the microscope.

Scanning Electron Microscopy, SEM (ZEISS- LEO SUPRA 55, Jena, Germany) was used to examine the morphology of the biogenic nanoparticles, where a fine gold coat was deposited on the samples under vacuum in a JFC-1100 sputter coater (JEOL) for 3 min at 15 mA. The elemental composition of the biogenic nanoparticles was measured using Energy-dispersive X-ray spectroscopy (EDX) onto a Bench Top SEM + EDX, JEOL, JCM 6000 plus, (Akishima, Tokyo, Japan), under EDX conditions of 15 kV and a working distance of 19 mm.

#### 2.6.3. Zeta potential and X-ray diffraction measurements

The Zeta potential of the biogenic and the commercial copper nanoparticles was determined using Dynamic Light Scattering (DLS) measurements (Malvern Zeta Sizer, Nano ZS, Malvern, UK), with a helium-neon laser operating at 90° scattering angle and 633 nm wavelength at 25°C.

An X-ray diffractometer with Cu Kα radiation (Bruker D8 Discover, Karlsruhe, Germany) at a wavelength of 1.54 A^0^ was used to examine the phase and constitution of the biogenic nanoparticle powders which were precipitated on a silicon holder.

### 2.7. Chemical analysis of the crude extract and the biogenic Cu NPs

Total carbohydrate contents of CE and the biogenic Cu NPs were determined by the phenol-sulfuric method using glucose as a standard. In addition, the total phenolic content was determined using the Folin-Ciocalteu reagent according to the method described by Singleton & Rossi [21, 22], with minor modifications [23] and using Gallic acid as a standard.

### 2.8. Testing the biological activities of the crude extract, the biogenic and the commercial Cu NPs

#### 2.8.1. Seed germination assay

This assay was conducted to investigate the effect of CE and Cu NPs (biogenic and commercial) on seed germination and seedling growth. Seeds of monocotyledonous wheat plant (*Triticum aestivum* L.) of the Egyptian Sakha 93 cultivar were collected and classified at the Agricultural Research Centre (ARC) in Egypt to be further examined. The test was carried out according to the ASTM standard germination protocol (ATSM 2003) with slight modifications [24]. The CE, biogenic and commercial Cu NPs were serially diluted with distilled water to the concentrations of 0.00 (control), 0.03, 0.22, and 0.43 mg/mL. Ten seeds of wheat were placed on Whatman filter papers No. 10 in Petri dishes (10-cm diameter), and three mL of each concentration were added per dish. Three replicates were used for each concentration such that the total number of tested samples were 30 samples/treatment. All dishes were covered with a parafilm and were incubated (Thermos Fisher incubator, Marietta, Ohio 45750, United States) at 25±2°C in the dark for 7 days; after which the root and shoot lengths were recorded. The dishes were monitored regularly during the incubation period to ensure they were kept wet. The relative elongation %, relative germination rate and germination index were calculated as follows:

$$Relative\;germination\;rate=\left[\frac{Seeds\;germinated\;in\;tested\;sample\;}{Seeds\;germinated\;in\;control}\right]*100$$ [1]

$$Relative\;shoot/root\;elongation=\left[\frac{Mean\;shoot/root\;length\;in\;tested\;sample\;}{Mean\;shoot/root\;length\;in\;control}\right]*100$$ [2]

$$Germination\;index=\left[\frac{Relative\;germination\;rate*relative\;root\;elongation\;}{100}\right]$$ [3]

#### 2.8.2. Antioxidant and oxidase enzymes assay for the crude extract and the biogenic Cu NPs

This assay was used to quantify the enzyme activity in the wheat seeds that were germinated in the petri dishes. The tests were only conducted for the seeds subjected to each of the CE and the biogenic Cu NPs because the commercial Cu NPs did not show a significant effect on the root and shoot growth as will be shown later.

*2*.*8*.*2*.*1*. *Preparation of a crude enzyme extract*. A crude enzyme extract was prepared by grinding wheat seedlings (500 mg) that are 3–4 days old in a mortar with 5 mL of cold Na/K phosphate buffer (0.1M) at pH 6.8. The homogenate was centrifuged for 20 min at 6000 rpm and 4°C. Then, the supernatant was used for measuring the activities of the following enzymes spectrophotometrically (Jenway UV-Vis spectrophotometer, model no. 6405, Market Harborough, LE169AF, United Kingdom). The enzyme activity was expressed as μg /g fresh wt min^-1^.

*2*.*8*.*2*.*1*.*1*. *Peroxidase assay (EC*.*1*.*11*.*1*.*17)*. Peroxidase activity was measured by Guaiacol method [25] with minor modifications. The 3-mL assayed mixture contained 2.2 mL of 0.1M potassium phosphate buffer (pH 7.0), 0.5 mL of 0.018 mM guaiacol, 0.2 mL of H_2_O_2_ (30%) and 0.1 mL of the crude enzyme extract. Color intensity was detected at 436 nm by recording the changes in absorbance within 30 s to 3 min.

*2*.*8*.*2*.*1*.*2*. *Catalase assay (EC*.*1*.*11*.*1*.*6)*. Catalase activity was measured using a hydrogen peroxide assay that is based on the formation of its stable complex with ammonium molybdate [26]. In this assay, 0.2 mL of the plant extract was incubated in a 1-mL reaction mixture containing 65 mM of hydrogen peroxide in 60 mM Na/K phosphate buffer, pH 7.4 at 25°C for 4 min. The enzymatic reaction was stopped with 1 mL of 32.4 mM ammonium molybdate, and the concentration of the yellow complex of molybdate and hydrogen peroxide was measured at 405 nm.

*2*.*8*.*2*.*1*.*3*. *Ascorbic acid oxidase (AAO) assay (EC*.*1*.*10*.*3*.*3)*. Ascorbic acid oxidase activity was measured according to a previously reported method [27]. To a quartz cuvette of a UV spectrophotometer, one mL of 0.2 M phosphate buffer (pH 6.2), 0.2 mL of 1 mM ascorbic acid and 0.2 mL of the enzyme extract were added. Distilled water was added to bring the final volume to 3.0 mL. The initial absorbance was recorded, and then the optical densities after 30 s to 3 min were measured in order to monitor the rate of disappearance of ascorbate at 265 nm.

*2*.*8*.*2*.*1*.*4*. *Polyphenol oxidase (PPO) assay (EC*.*1*.*10*.*3*.*1)*. Polyphenol oxidase activity was determined according to a previously reported method [28]. The assayed mixture constituted 3 mL of buffered catechol solution (0.01 M catechol, freshly prepared in 0.1 M phosphate buffer of pH 6.0) along with one mL of the enzyme extract. Changes in absorbance were recorded every 30 s to 5 min using a UV spectrophotometer at 495 nm.

#### 2.8.3. Nanoparticles uptake in shoot and root of wheat seedlings

The copper uptake of the wheat seedlings germinated in the petri dishes and subjected to each of the biogenic and the commercial Cu NPs was determined using a Microwave Plasma Atomic Emission Spectrometer (MPAES, Agilent, Santa Clara, CA 95051, United States). The shoot and root ash were separately placed into digestion vessels containing a mixture of 1: 0.2 of HNO_3_-H_2_O_2_ by volume. These vessels were heated to 85°C for 90 min to ensure digestion of the ash, then filtered through a 0.45-μm nylon sealed filter membrane. The filtered sample was used for (MPAES) analysis in shoots and roots at different concentrations of the biogenic and the commercial Cu NPs.

The translocation factor (TF) was used to evaluate the translocation of copper from wheat root to shoot [29]. This was calculated as the ratio of Cu concentration in wheat shoot to that in plant root:

$$TF=\left[\frac{C\;plant\;shoot}{C\;plant\;root}\right]$$ [4]

#### 2.8.4. Pot experiment

**Fig 2:**
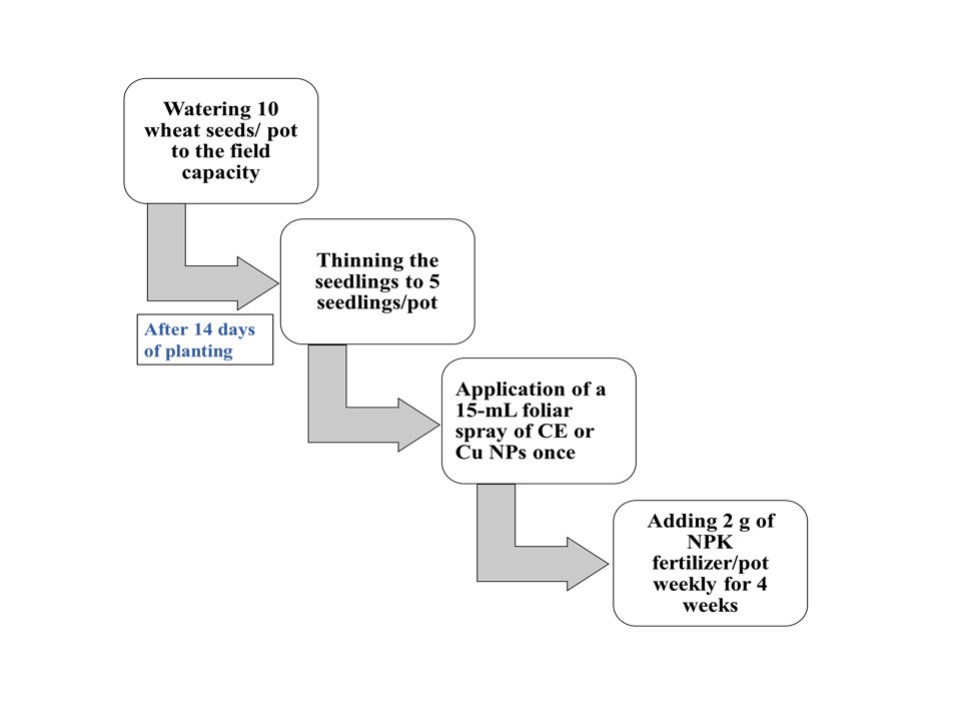
Schematic outline of the pot cultivation. Physical and chemical characteristics of the soil used in this study as well as the procedure used for Chlorophyll extraction and estimation of chlorophyll a, b and total chlorophyll contents were previously reported by Essa et al. [20].

In a silt clay soil, Sakha 93 wheat seed (*Triticum aestivum L*.) was planted during the 2018/2019 Winter season. It was thinned to 5 seedlings per pot. After 14 days of planting, a foliar spray of CE or Cu NPs was applied while irrigating the soil to keep it at almost its field capacity for the growing season period. After 15 days from seedling, 2 g of NPK fertilizer were added per pot area, since the rate of application of NPK fertilizer is 1 kg per 1000 m^2^ of the field. NPK constitutes (20/20/20) by weight (on a dry basis) of Nitrogen/ Phosphorus/ Potassium elements. The process was repeated every week throughout the period of the experiment (4 weeks). Foliar treatment was applied using each of CE and Cu NPs, with concentrations of 0.00 (control), 0.06, and 0.43 mg/mL, along with a treatment volume of 15 mL. Fig 2 shows the steps of the pot cultivation process.

### 2.9. Statistical analysis

The statistical analysis was performed using SPSS program, version 20 for Windows (SPSS Inc, USA). A one-way analysis of variance (ANOVA) was conducted to test the significant differences (*p* < 0.05) between the treatment and control means. Duncan’s multiple range test (DMRT) was also performed to determine the significant differences between the tested groups.

## 3. Results and discussion

### 3.1. Chemical and phytochemical analyses

Chemical analysis of the CE and the biogenic Cu NPs showed that they have carbohydrate contents of 50.3 ± 3.1% and 35.6 ± 3.2%, as well as phenolic contents of 27.5 ± 0.1 and 3.13 ± 0.05 mgGA/g, respectively. Obviously, the NPs have lower contents since they are capped by only a layer of the extract. The carbohydrate content of CE was further confirmed by GC-MS analysis as shown in S1 Fig and Table 1, where CE mainly constitutes the monosaccharaides of D-mannose (29.21%), D-fructose, (18.51%), L-sorbose (12.91%), D-galactose (5.47%) and D-Talose (5.21%). To the best of our knowledge, this is the first report on the quantification of sugars of *Avicennia marina* by GC/MS.

**Table 1:** Identified contents of the crude extract (CE) as measured using metabolite profiling on GC/MS.

Peak no.	rt* (min)	m/z**	Area (%)	Component
**M1**	4.6648	73	11.410	Propionic acid
**M2**	5.5986	147	9.118	Oxalic acid
**M3**	9.5346	205	5.963	Glycerol
**M4**	23.0428	217	18.510	D-Fructose
**M5**	23.2624	307	12.914	L- (-)-Sorbose
**M6**	23.3852	319	2.196	Ribitol
**M7**	23.5277	205	29.205	D-Mannose
**M8**	23.8882	73	5.473	D-Galactose
**M9**	23.8974	147	5.207	D-(+)-Talose

*retention time

**mass to charge ratio.

### 3.2. Synthesis and characterization of the biogenic Cu NPs

**Fig 3:**
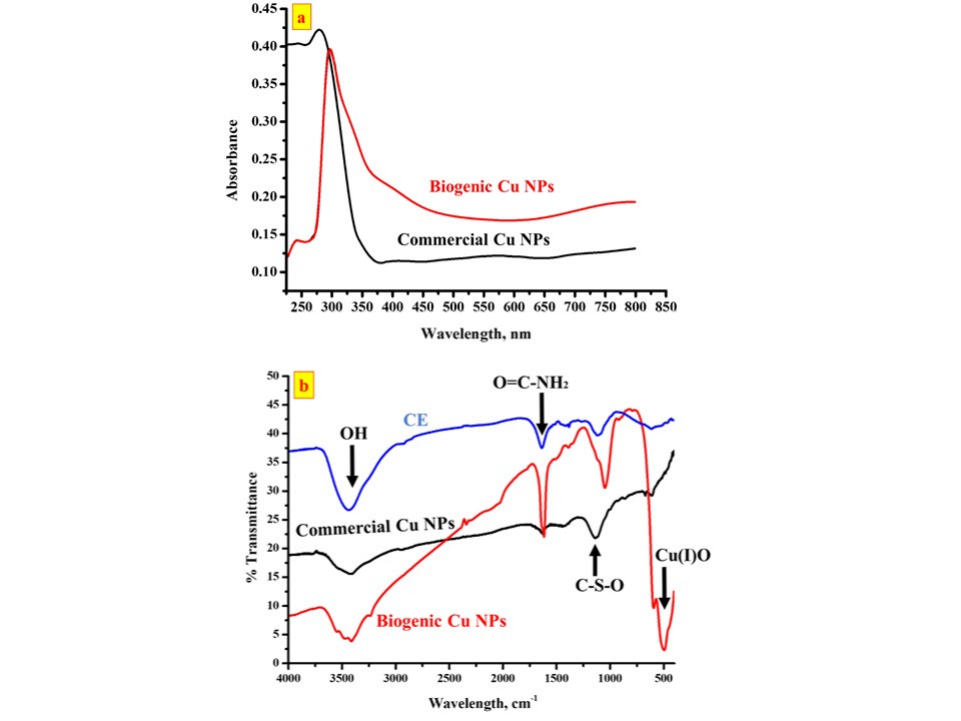
UV-Vis spectra of the biogenic and commercial Cu NPs (a), together with the FTIR spectra of the CE, biogenic and commercial Cu NPs (b).

The formation of the biogenic Cu NPs was confirmed by UV-Vis spectroscopy and FTIR spectroscopy as depicted in (Fig 3A and 3B). Fig 3A shows the UV-visible spectra of the biogenic Cu NPs along with their commercial counterparts. Both spectra exhibit an absorbance peak at the range of 290–320 nm which is in good agreement with the previous literature reported for Cu NPs synthesized through bio-reduction [30, 31]. To determine the functional groups present in the layer capping the biogenic NPs, the FTIR spectrum of the NPs was analyzed then compared to the spectra of CE and the commercial Cu NPs as depicted in Fig 3B. Clearly, CE and both Cu NPs show bands in the range of 3500–3400 cm^-1^, corresponding to the stretching vibration of the bound hydroxyl (O-H) groups. In addition, CE exhibits a band in the range of 1690–1630 cm^-1^ that could be attributed to the carbonyl stretch of the amide group, as well as two peaks in the ranges of 1385–1380 cm^-1^ and 1200–1000 cm^-1^ that could be ascribed to ester sulfate and acidic polysaccharides, respectively. Furthermore, the appearance of the vibration band of Cu_2_O on the spectrum of the biogenic Cu NPs at 670 cm^-1^ confirms the formation of Cu (I) oxide nanoparticles [32]. On the other hand, the vibration band of CuO shown on the spectrum of commercial Cu NPs at 490 cm^-1^ indicates the presence of Cu (II) oxide nanoparticles [32]. Comparing the spectra of CE and the biogenic Cu NPs, it can be deduced that most of the peaks that were clearly exhibited by CE diminished in the Cu NPs implying that most of the functional groups present in CE contributed to form the stabilizing layer onto the biogenic Cu NPs. Previous studies reported that carbonyl groups from amino acid residues and proteins have a strong ability to bind to metals and hence could contribute along with the polysaccharides in capping the nanoparticles [33].

**Fig 4:**
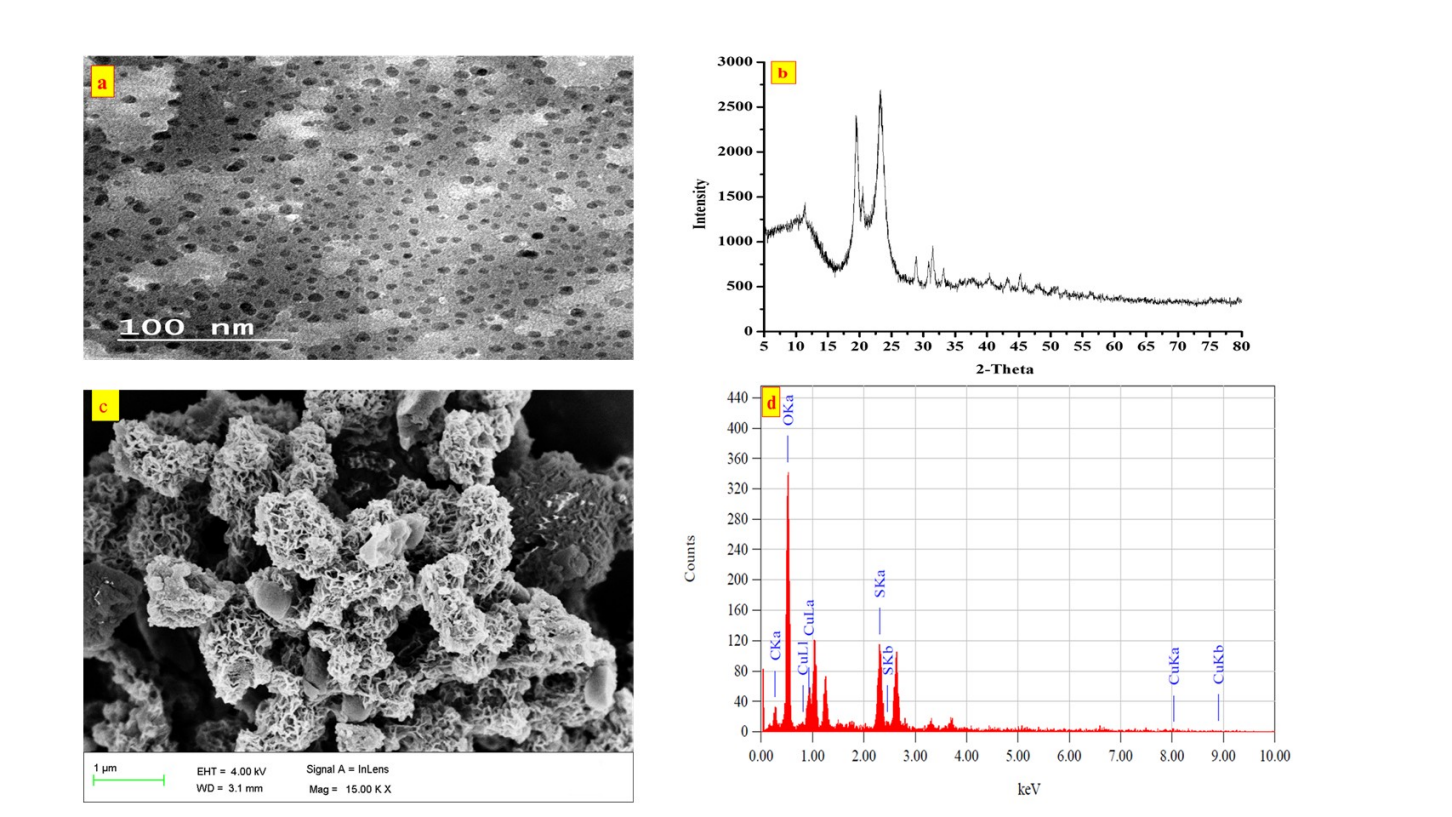
TEM image (a), XRD pattern (b), SEM image (c), EDX analysis (d) and of the biogenic Cu NPs.

The composition and nano structure of the biogenic Cu NPs were examined by TEM, XRD, SEM, and EDX as depicted in (Fig 4A–4D). The TEM micrograph of the biogenic Cu NPs (Fig 4A) reveals regular homogeneous spherical shaped particles with an average diameter of about 11.60 ± 4.65 nm which is similar to values reported in previous literature [34]. The particles have a sponge like morphology with large surface area as shown in their SEM micrograph (Fig 4C). The EDX elemental composition of the biogenic Cu NPs (Fig 4D) constitutes 47.80% O, 8.86% C, 27.01% S and 16.34% Cu by mass. To determine the Cu phases present in the structure of the NPs, XRD patterns (Fig 4B) of the biogenic and commercial Cu NPs were studied. As clear from the spectrum of the biogenic Cu NPs, peaks are displayed at approximately 11.46°, 18.1°, 22.9°, 32° and 46° and hence could be attributed to a mixture of Cu species [35–37], mainly the orthorhombic phase of Cu(OH)_2_ (JCPDS: #80–0656), and the cubic phase of Cu_2_O (ICCD-JCPDS: #78–2076) which correspond to the crystallographic planes of reflection of (020), (021), (111) and (200), respectively. Based on the presence of the two major peaks of 18.1° and 22.9°, the constituent ratio of Cu_2_O to Cu(OH)_2_ was estimated from the relative percentage of each peak to be 2:1. Since the pH of the nano solution was 6–7 and according to Pourbaix diagram, copper oxide would more likely exist in the form of Cu (I) species. Dynamic light scattering (DLS) measurements showed that the biogenic and commercial Cu NPs are nearly neutral with a zeta potential of 1.02 and 0.02 mV, respectively at the working pH range of 6–7.

### 3.3. Wheat seedlings root and shoot lengths

**Fig 5:**
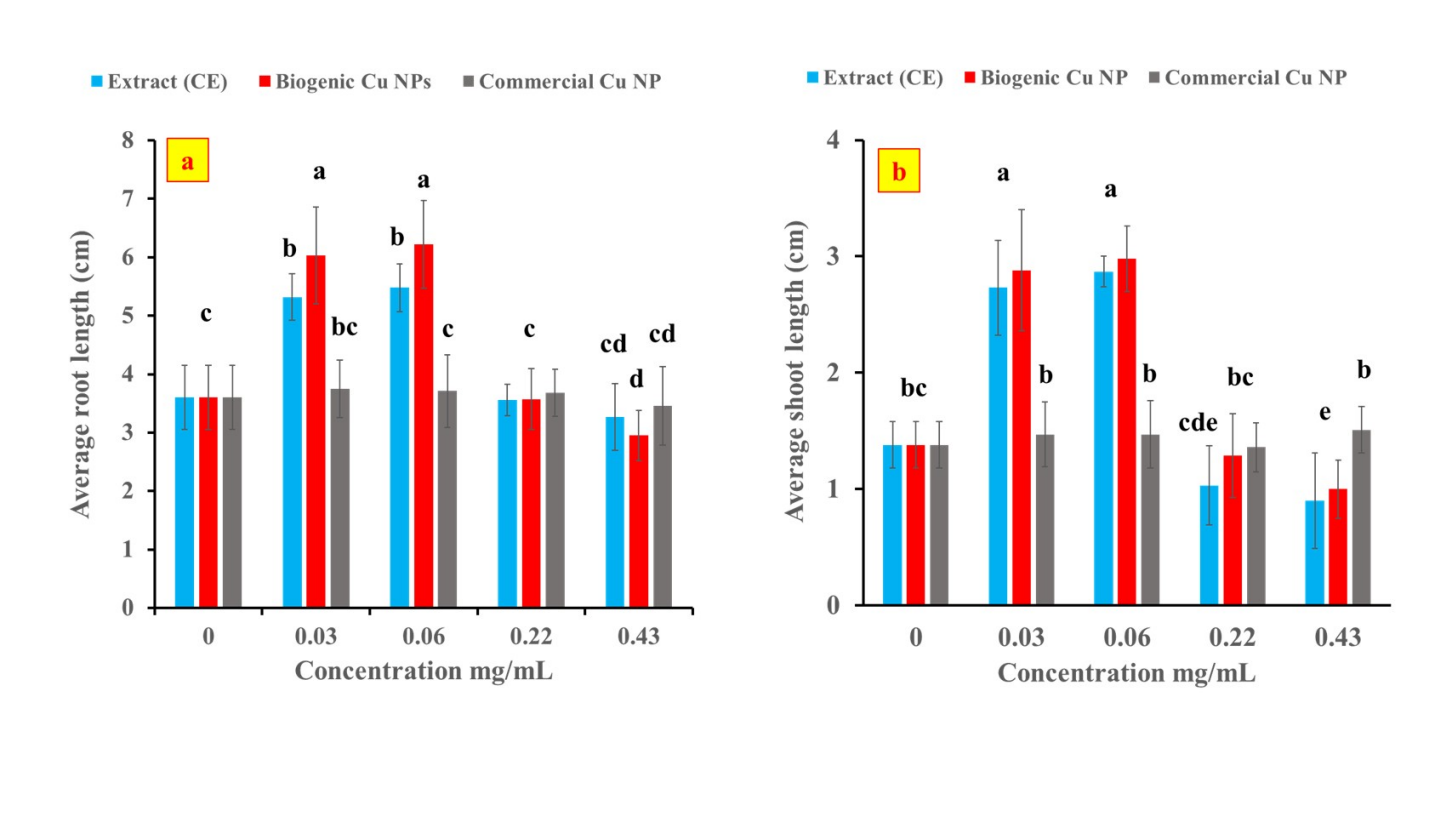
Root (a) and shoot lengths (b) of the germinated wheat seeds after their incubation in CE, biogenic and commercial Cu NPs.

Fig 5A and 5B show the respective average root and shoot lengths of the germinated wheat seeds (incubated in the petri dishes at 25±2°C in the dark for 7 days) after their exposure to CE, biogenic and commercial Cu NPs. Relative to the control, both CE and the biogenic Cu NPs significantly enhanced the root and shoot lengths of wheat seedlings when applied at the lower concentrations of 0.03 and 0.06 mg/mL. At these concentrations, the biogenic Cu NPs had a stronger stimulant effect than that of CE on the root length of wheat seedlings, while it showed a comparable effect to that of CE on the shoot length. In this respect, the root length was promoted by 47.77and 52.22% relative to the control at 0.03 and 0.06 mg/mL of CE, respectively; as opposed to 67.50 and 72.77% at the respective concentrations of the biogenic Cu NPs. However, the effect was not signficant on the root and shoot lengths of the seedlings upon their treatment with 0.22 mg/mL. Upon exposure to 0.43 mg/mL of CE, the root length was unaffected while the shoot length decreased by 34.78% relative to the control. A previous study revealed that root elongation of *Vulgaris phaseolus* was promoted upon exposure to low concentrations of 25-nm copper oxide nanoparticles [38]. The biogenic Cu NPs is the only tested material that significantly inhibited the root and shoot lengths of wheat seedlings when applied at the highest concentration of 0.43 mg/mL. This inhibitory behavior was previously reported for copper nanoparticles (40–50 nm) that were applied on wheat seeds [39].

This dose dependent effect is in agreement with previous studies which recorded a significant increase in root length of wheat seeds upon their exposure to low concentrations of Cu NPs biosynthesized from *Moringa oliefera*, meanwhile an adverse effect was realized at higher concentrations of Cu NPs that exceeded 0.075 mg/mL [40]. In addition, a similar dose dependent effect has been reported in a previous study that examined the effect of copper oxide nanoparticles (< 50 nm) on root and shoot elongation of soybean and chick pea seeds [15], as well as another study that reported the impact of biogenic cuprous oxide nanoparticles (Cu_2_O NPs) on *Lycioersicum esculentum* tomato seedling growth [41].

As for the commercial Cu NPs, they did not exhibit any significant effect on the root and shoot lengths of wheat seedlings at all the applied concentrations. This behavior may be owed to the difference in the oxidation states between the cupric oxide commercial Cu NPs (oxidation state: +2) and the primarily cuprous oxide biogenic ones (oxidation state: +1). It is to be noted here that the biogenic nanoparticles constitute mainly Cu (I) oxide as confirmed earlier by XRD. In addition, the commercial Cu NPs do not have a capping layer of active functional groups as in the biogenic Cu NPs. Furthermore, the biogenic Cu NPs have additional Cu(OH)_2_ species which might have enhanced the penetrability of the nanoparticles into the root [42].

### 3.4. % Relative shoot and root lengths and germination index of wheat seedlings

**Fig 6:**
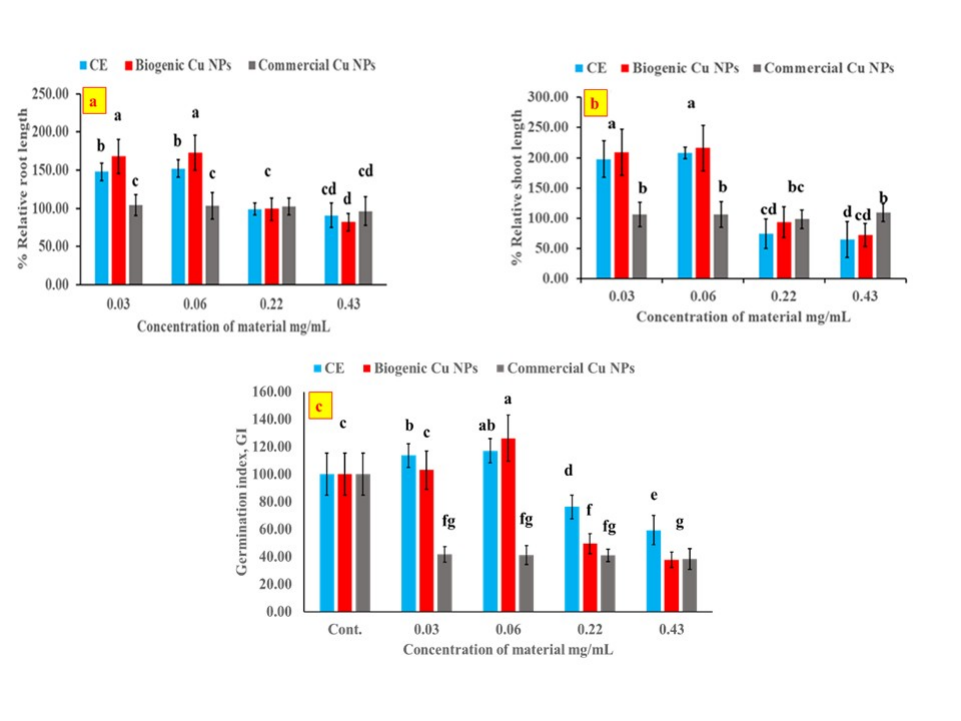
Effect of CE, biogenic and commercial Cu NPs on % relative growth of root (a) and shoot lengths (b) and germination index of wheat seeds (c).

Data pertaining to percent relative growth in shoot and root lengths of the petri dish- germinated wheat seeds relative to the control is shown in (Fig 6A and 6B). Obviously, the CE and the biogenic Cu NPs had an adverse effect on both shoot and root growth when applied at 0.22 and 0.43 mg/mL. The relative growth % of shoot and root decreased gradually with increasing the concentration of the CE and the biogenic Cu NPs, recording their respective lowest values of 65.22 ± 29.90 and 72.46 ± 18.78% alongside with 90.83 ±16.09 and 81.94 ± 11.70%, at the highest employed concentration of 0.43 mg/mL CE or biogenic Cu NPs, respectively. Commercial Cu NPs, on the other hand, showed no significant effect on both shoot and root growth. Fig 6C depicts the germination index (GI) of wheat seedlings, which showed its respective highest values of 117.09 and 123.61 upon exposure to the CE and the biogenic Cu NPs at 0.06 mg/mL. At the highest applied concentration of 0.43 mg/mL for the CE and the biogenic Cu NPs, GI recorded 59.39 and 37.82, respectively. Again, commercial Cu NPs had no significant effect on the germination index of wheat seedlings due to the reasons alluded to earlier.

From the above-mentioned results, it can be deduced that the biogenic Cu NPs could have both positive and negative effects on seed germination and growth depending on the concentration at which they are applied. This behavior was also observed with silver nanoparticles that were applied to 11 species of wetland plant [43], while the reduction in germination index with increasing the concentration of Cu NPs was also reported in previous work on wheat seeds [44]. Similar inhibitory effects were observed on rice (*Oryza sativa*) (monocotyledonous), when treated with copper oxide nanoparticles (< 50 nm) in both stages of seed germination and seedling growth due to the accumulation of Cu in root and leaf tissues. This was indicated by the massive increase of Cu in roots (76–fold) and leaves (5.5–fold) in comparison with the control [45], or the expression of OsCYCD and OsCDC2 genes which inhibited growth [46], or alternatively significant reduction in lengths and weights of shoot and root [47]. In addition, the adverse effect of Cu NPs could have been caused as a result of the oxidative stress induced by the NPs due to their interference with the electron transport system, affecting the biochemical pathways in the seeds through oxidation of proteins, lipids and nucleic acids, or via changing in the phytohormones which alter plant metabolism, and/or through regulation of reactive oxygen species (ROS) by producing antioxidant enzymes for protection of the cellular and sub-cellular system from these cytotoxic effects [45, 46]. Several factors could influence the toxicity of Cu NPs, including their applied concentration, particle size, specific surface area, shape, stability and its physiochemical characteristics. Other physiological and morphological factors related to the plant species could also be at play such as plant type, age, life cycle, in addition to external growth factors as growth media, and diluting agents [48–51]. All these factors will dictate the extent of accumulation and uptake capacity of the NPs onto the plant. The stimulatory effect of Cu NPs, on the other hand, could be owed to the transfer of some NPs to the meristem where intensive cell division occurs. Thus, cell growth was promoted. This is also supported by a study which showed that Cu NPs induce the modulation of auxin related genes which play a significant role during the growth of apical meristems [46, 52].

### 3.5. Detoxification enzymes activity in the petri dish-germinated wheat seedlings

**Fig 7:**
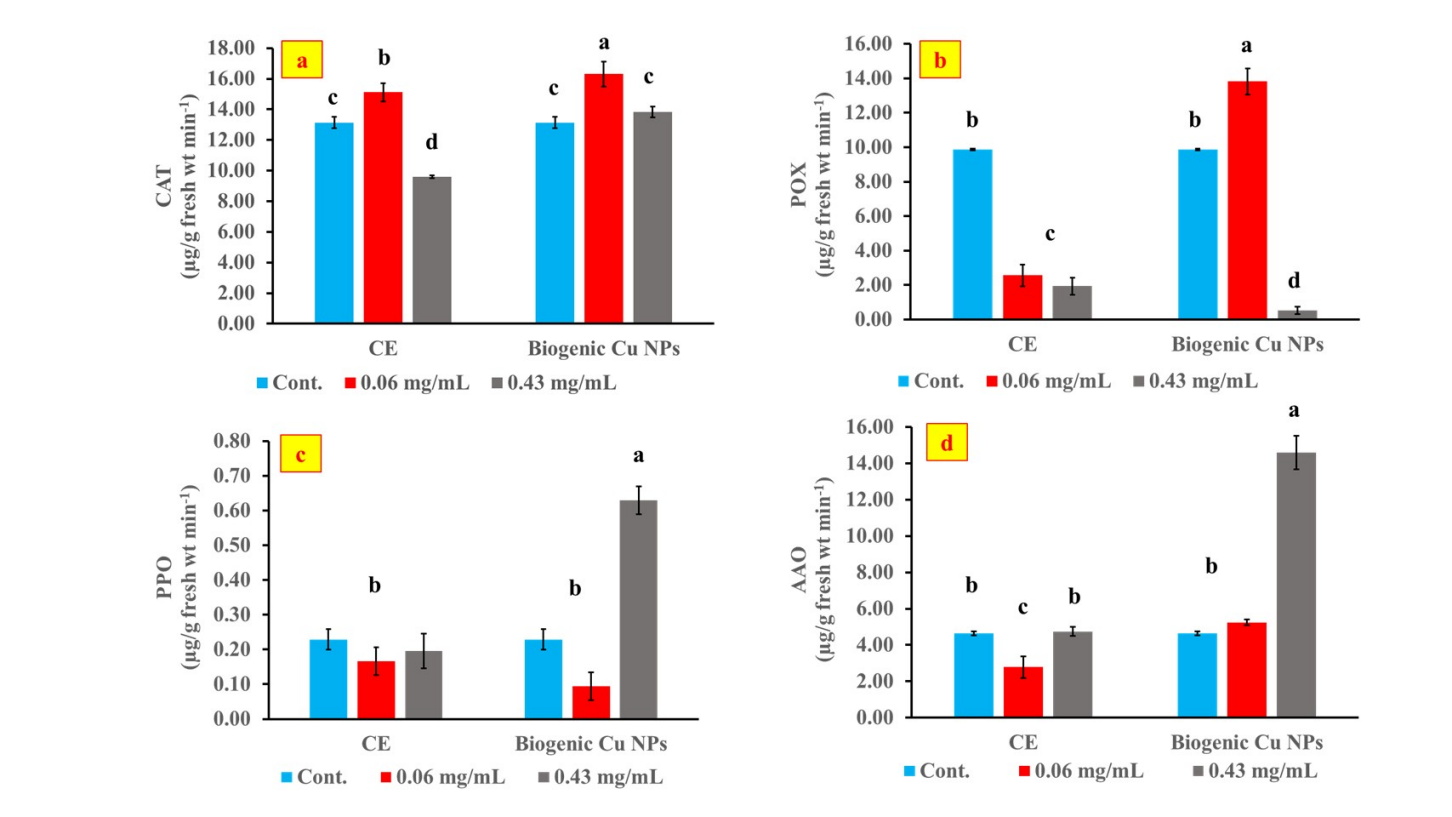
Effect of CE and Cu NPs on CAT (a), POX (b), PPO (c) and AAO (d) activities in wheat germinated seeds (μg/g fresh wt min^-1^) at the 4^th^ day of germination.

To gain more insight into the possible mechanism of inhibition, the effect of each of the CE and the biogenic Cu NPs on the antioxidant and oxidase enzyme activities of peroxidase, catalase, ascorbic acid oxidase, and polyphenol oxidase enzymes in wheat seeds was investigated. (Fig 7A–7D) shows the antioxidant activities after applying the CE and the biogenic Cu NPs on wheat seeds, once in a low concentration of 0.06 mg/L and again at a high concentration of 0.43 mg/mL. These two concentrations were chosen since the former stimulated the plant growth while the latter inhibited it. Commercial Cu NPs, however, were not tested as they did not show any significant effect on the seedlings growth and the germination index of the wheat seeds as discussed earlier.

Peroxidase (POX) and catalase (CAT) are antioxidant enzymes that can detoxify H_2_O_2_ and scavenge free radicals and oxygen intermediates [53]. As clear from Fig 7A, applying the low concentration (0.06 mg/mL) of the CE or the biogenic Cu NPs to wheat seeds significantly (P < 0.05) increased CAT activity relative to the control. In addition, the effect of the biogenic Cu NPs is more pronounced than that of the CE at the low concentration. This behavior is consistent with the promotion in root and shoot growth observed at this concentration. On the other hand, applying the high concentration (0.43 mg/mL) of CE reduced the activity, while employing the same concentration of Cu NPs did not significantly affect the activity relative to the control. This again is in agreement with the inhibition of root and shoot exhibited at this concertation. A similar dose-dependent effect was previously reported for TiO_2_ NPs on CAT enzyme during the seed germination of onion [54]. As for POX activity shown in Fig 6B, it decreased significantly relative to the control upon the application of either the low or high concentration of CE, or the high concentration of Cu NPs. A similar inhibitory effect for chitosan NPs in reducing POX activity of broad beans was previously reported [55].

The effect of each of the CE and the biogenic Cu NPs on the activities of PPO and AAO enzymes is shown in (Fig 7C and 7D). The high concentration of Cu NPs (0.43 mg/mL) significantly (P < 0.05) enhanced PPO and AAO activities in the wheat germinated seeds as compared to the control (Fig 7C and 7D), while the same concentration for CE did not significantly affect the activities. This finding is in line with the seed germination results which showed inhibition in seedling growth at the higher concentration of 0.43 mg/mL Cu NPs. The phenolics oxidation reaction is enhanced by PPO in plants, and the relation between phenolic content and PPO activity was reported to be inversely proportional in many plants, such as watermelon and tomato plants subjected to heat and cold stresses [56]. Hence, increasing PPO and AAO activities can result in decreasing the antioxidant activity and this might consequently lead to growth inhibition.

### 3.6. Copper uptake and translocation factor in the petri dish-germinated wheat seedlings

**Fig 8:**
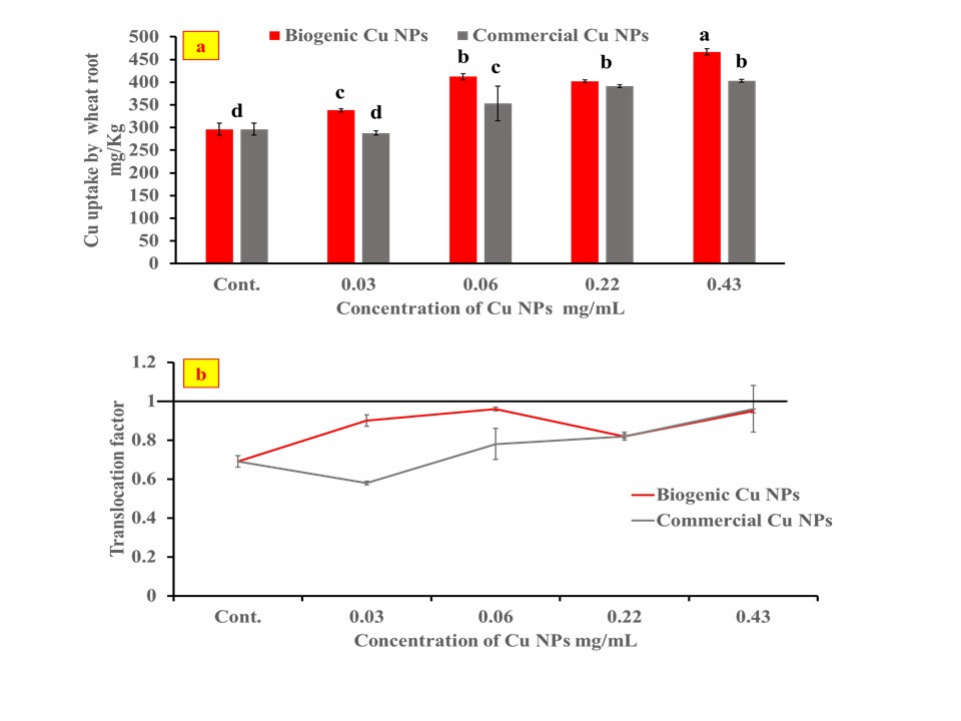
Cu uptake in wheat seedlings (a) and Translocation factor of wheat seedlings (b).

Fig 8A depicts the copper uptake in root tissue at different applied concentrations of the biogenic and the commercial Cu NPs. Relative to the control, the copper uptake increased with increasing the concentration of the biogenic as well as the commercial Cu NPs. It is to be noted that the copper detected in the control is the copper mineral already present in the plant [57]. A respective increase of 1.57 and 1.36–fold in the Cu content was observed in the root treated with 0.43 mg/mL of the biogenic and the commercial Cu NPs as compared to the control. This finding is in agreement with a previous study conducted on copper oxide nanoparticles taken up in *Oryza sativa* leaves [58]. The copper uptake in roots treated with 0.43 mg/mL of the biogenic Cu NPs (467.06 ± 6.89 mg/kg) is greater than that observed in roots treated with the commercial Cu NPs (403 ± 3.48 mg/kg). This might explain the enhancement in root lengths observed earlier (Fig 6A and 6B) in addition to the stimulation in the activities of the antioxidant enzymes of CAT and POX (Fig 7A and 7B). Previous work reported that copper is a necessary element for plant growth (micronutrient), since it stimulates numerous enzymes and has a role in the synthesis of RNA, therefore it promoted the seedlings growth at a less than 50 μM concentration [59]. Other studies attributed the stimulation of seed germination by NPs to the increase in the expression levels of a gene encoding catalase, and suggested that CAT is the vital enzyme that controls the recovery of seed vigor in aged seeds [60]. On the other hand, applying the higher concentration of 0.43 mg/mL of the biogenic Cu NPs significantly decreased shoot and root lengths due to the higher accumulation of Cu in the roots as well as the higher PPO and AAO enzyme activities (Fig 6C and 6D) obtained relative to the control.

The translocation of copper from wheat root to shoot is shown in (Fig 8B). Obviously, TF of Cu was less than unity (<1) in all treatments which explains the lower concentrations of Cu observed in shoots than roots as shown in (Fig 8B). The root has the ability to accumulate some metals to protect the aerial parts (edible parts) besides its main functions of fixing the plant in the soil and absorption of water and dissolved minerals [61].

### 3.7. Chlorophyll contents in the wheat leaves treated with foliar spray

**Fig 9:**
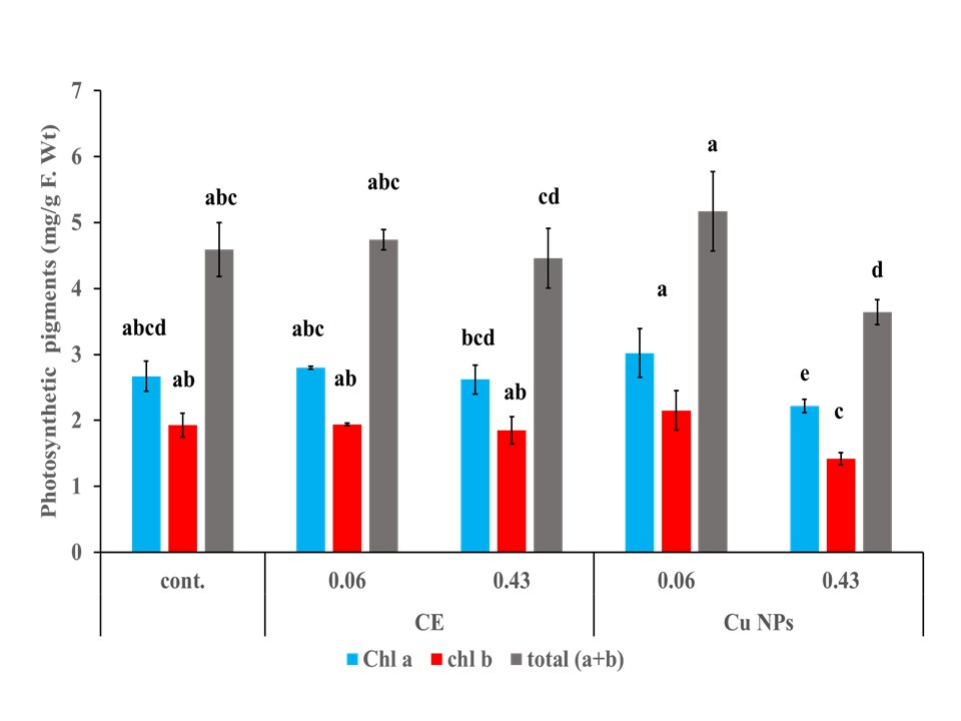
Chlorophyll a, b and total chlorophyll contents upon exposure to CE and Cu NPs.

Fig 9 shows the contents of chlorophyll a, b and total chlorophyll in wheat leaves upon their treatment with a foliar application of CE and biogenic Cu NPs. As clear from the figure, chlorophyll a, b and total chlorophyll contents of CE were insignificantly changed as compared to the control at a concentration of 0.06 mg/mL, recording 2.80, 1.94, and 4.74 mg/g fresh weight after 21 days from application. A previous study has reported similar trends where the effect of low concentrations of cyanobacterial extracts on chlorophyll b of lettuce plants was examined during two different seasons [62]. Similarly, the treatment with biogenic Cu NPs followed the same behavior as that of CE at the same concentration resulting in 3.02, 2.15, and 5.17 mg/g fresh weight of chlorophyll a, b, and total chlorophyll contents, respectively after 21 days from application. This behavior resembles that previously reported for the effect of Fe_3_O_4_ nanoparticles (25 nm) on chlorophyll (a) and total chlorophyll contents of Rocket (*Eruca sativa*) at concentrations of 1 and 2 mg/L after 5 weeks from the application [63].

On the other hand, the previous growth parameters in wheat leaves decreased insignificantly at the highest concentration of 0.43 mg/mL of CE. Meanwhile, treatment with biogenic Cu NPs showed an inhibitory effect on chlorophyll a, b, and total chlorophyll at the same concentration since it reduced the chlorophyll a, b and total chlorophyll contents relative to the control by 16.85, 26.42, and 20.69%, respectively. The inhibitory effect of Cu NPs was previously examined when studying the impact of 1 mg/L of CuO nanoparticles on total chlorophyll content, as well as chlorophyll (a) and (b) of *Arabidopsis thaliana* seedlings [64].

This current study of biogenic Cu NPs highlights the dose-dependent effect of the nanoparticles in both seed germination and foliar application. Similar findings were reported for the effect of copper oxide nanoparticles on seedlings lengths, germination percentage, and chlorophyll contents of soybean (*Glycine max* (L.) [65]. Racuciu and Creange [66] found that the content of chlorophyll in maize plants was enhanced at a low concentration of Ag NPs (10–50 μL/L), but the chlorophyll content was reduced with a high concentration treatment of NPs. In addition, Gohari et al. [67] found that the high concentration (i.e, 200 mg/L) of titanium dioxide nanoparticles (TiO_2_ NPs) decreased the chlorophyll content of *Dracocephalum moldavica* seedlings as opposed to the lower concentration (i.e, 100 mg/L), which enhanced the pigment content.

The nanoparticles were proposed to have the ability to penetrate the coat of the seeds and enhance the uptake and use of water. Consequently, the enzyme systems would be stimulated, and the seed germination and growth would be improved [68]. Additionally, Suriyaprabha et al. [69] explained the role of nano-SiO_2_ in enhancing maize germination by providing better growth conditions such as nutrients, pH, and conductivity. In another study, Lu et al. [70] owed the enhancement in soybean seed germination by nano-SiO_2_ and nano-titanium dioxide (nano-TiO_2_) to the improvement in the nitrate reductase activity. This result was confirmed by Yang et al. [71] and Mishra et al. [72] who found that TiO_2_ NPs controlled the activity of the nitrogen metabolism enzymes. Nitrate reductase, glutamate dehydrogenase, glutamine synthase, and glutamic-pyruvic transaminase supported the plants’ uptake of nitrate and aided in the conversion of inorganic nitrogen to organic nitrogen which forms protein and chlorophyll, and this probably increased the fresh and dry weights of the plant. In addition, the nanoparticles of metals can stimulate the efficacy of photosynthetic systems and their chemical energy production, since the chlorophyll in the reaction center combines with metal nanoparticles as Ag or Au, forming a unique hybrid system. This new system may produce more excited electrons by plasmon resonance [73].

However, the tendency of the NPs to penetrate walls and interact with cellular structures is owed to their properties as high reactive surfaces and small size. Therefore, they provide cellular capacity and genetic toxicity by oxidative stress introduction [74]. The inhibitory effect exhibited by the high doses of nanoparticles may be related to their phytotoxicity, which is correlated to the dissolution of toxic ions from the NPs [75], production of radicals through NPs reactions with plants, or direct reactions of NPs with plants [76]. Previous work also reported that Cu nanoparticles inhibit the growth of root hairs; these being important for the uptake of immobile nutrients such as phosphorus [74]. To sum up, plant nanotoxicity may depend on the dose, species of plant, and treatment conditions (substrate, temperature, and environment).

## 4. Conclusion

Biogenic Cu NPs of Cu(OH)_2_ and Cu (I) oxide species were successfully synthesized using the crude aqueous extract of *A*. *marina*. The nanoparticles exhibited a dose-dependent effect on the growth of wheat seedlings. Applying low concentrations of the biogenic Cu NPs onto the seedlings enhanced their growth, while treating the seedlings with high concentrations of the nanoparticles suppressed the seedling growth relative to the control. At 0.43 mg/mL, the root and shoot growth was inhibited relative to the control by 81.94 ± 11.70 and 72.46 ± 18.78%, respectively. Further in foliar application, the chlorophyll content was either enhanced or inhibited depending on the concentration of Cu NPs which was applied on the wheat leaves (21-days old). Low Cu NPs concentration (0.06 mg/mL) had the tendency to improve the chlorophyll content but the higher one (0.43 mg/mL) suppressed it. These results are consistent with those of seedling growth. Copper uptake by the petri-dish germinated wheat seedlings was also dose dependent; and it reached its maximum value, 467.06 ± 6.89 mg/kg at the highest applied concentration of the biogenic Cu NPs (0.43 mg/mL) with a translocation factor of less than unity indicating the accumulation of Cu in the root. The impact of the biogenic Cu NPs on seedling growth was compared to that of the commercial Cu NPs which showed no significant effect on the growth of shoot or root relative to the control. This was attributed to the difference in the nature of the Cu species present in the biogenic and commercial nanoparticles. The inhibition of wheat seedling growth by the biogenic Cu NPs may be related to the activity of the antioxidant enzymes of catalase, peroxidase, ascorbic acid oxidase and polyphenol oxidase and their role in the antioxidant detoxification. The phytotoxicity of the biogenic Cu NPs on seed germination and plant growth, when applied at a high concentration, suggests their potential to be utilized as natural herbicides whose action can be promoted by inhibition of the peroxidase enzyme activity and enhancement of the ascorbic acid oxidase and polyphenol oxidase activities. However, they can also be used to stimulate seedling growth when applied at low concentrations.

## Supporting information

S1 Fig Representative GC/MS chromatogram for the crude extract. (DOCX)

S1 Table Shoot copper content in wheat seedlings. (DOCX)
