# Supplementary figures and images for: Biogenic copper nanoparticles from Avicennia marina leaves: Impact on seed germination, detoxification enzymes, chlorophyll content and uptake by wheat seedlings
Source: PLoS One. 2021 Apr 15;16(4):e0249764. doi: 10.1371/journal.pone.0249764 (PMC8049258; doi:10.1371/journal.pone.0249764)

**
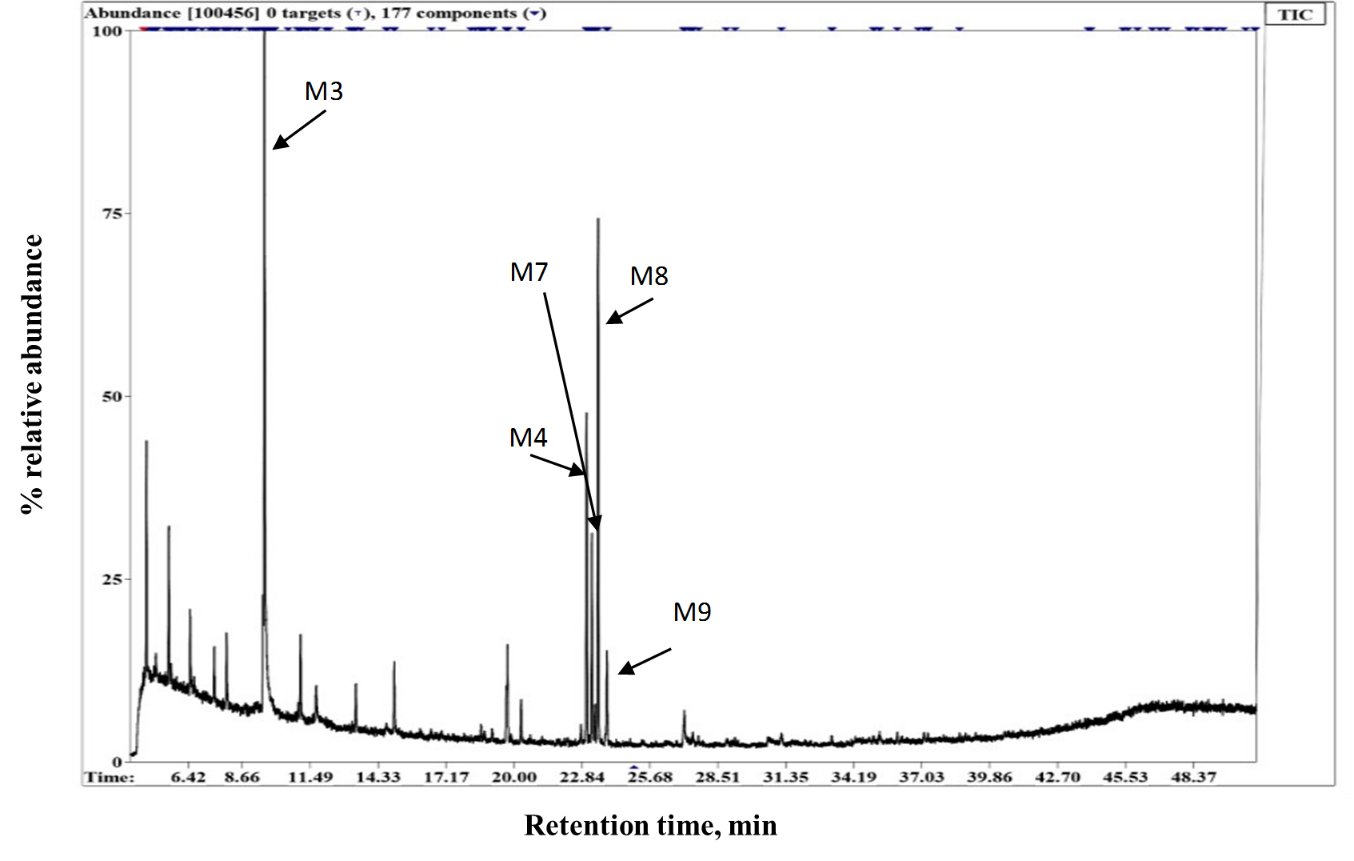
**

**S1 Fig.** Representative GC/MS chromatogram for the crude extract.

Supplement: S1 Fig — (DOCX) [file pone.0249764.s001.docx]
