# Supplementary material for: Biogenic copper nanoparticles from Avicennia marina leaves: Impact on seed germination, detoxification enzymes, chlorophyll content and uptake by wheat seedlings
Source: PLoS One. 2021 Apr 15;16(4):e0249764. doi: 10.1371/journal.pone.0249764 (PMC8049258; doi:10.1371/journal.pone.0249764)
Supplement: S1 Table — (DOCX) [file pone.0249764.s002.docx]

**S1 Table. Shoot Cu content**

| Concentration mg/mL | Shoot Cu content |
| --- | --- |
| Cont. | 424.20^d^± 2.94 |
| 0.03 | 494.67^a^ ± 6.19 |
| 0.06 | 448.80^c^ ± 2.30 |
| 0.22 | 473.87^b^ ± 2.13 |
| 0.43 | 416.86^e^ ± 1.79 |
